# Effects of Different Plasticizers on the Structure, Physical Properties and Film Forming Performance of Curdlan Edible Films

**DOI:** 10.3390/foods13233930

**Published:** 2024-12-05

**Authors:** Ying Chen, Jing Wang, Liang Xu, Yuping Nie, Yunyue Ye, Jianya Qian, Fengsong Liu, Liang Zhang

**Affiliations:** 1 School of Food Science and Engineering, Yangzhou University, Yangzhou 225127, China; 008265@yzu.edu.cn (Y.C.);; 2 Guangxi Key Laboratory of Clean Pulp & Papermaking and Pollution Control, School of Light Industry and Food Engineering, Guangxi University, Nanning 530004, China; 3 Guangdong Provincial Key Laboratory of Intelligent Food Manufacturing, Foshan University, Foshan 528225, China

**Keywords:** curdlan, edible film, plasticizer, hydrophobicity, mechanical properties

## Abstract

This study successfully developed edible films with excellent mechanical strength and notable water resistance, utilizing curdlan (CL) as the primary matrix and incorporating various plasticizers, including glycerol (GLY), ethylene glycol (EG), propylene glycol (PRO), xylitol (XY), sorbitol (SOR), and polyethylene glycol (PEG). A comprehensive suite of analytical techniques, including Fourier transform infrared spectroscopy (FTIR), wide-angle X-ray diffraction (XRD), scanning electron microscopy (SEM), dynamic mechanical analysis (DMA), and tensile testing, were employed to evaluate the films’ structural and mechanical properties. After incorporating PEG, the water sensitivity increased slightly, with a contact angle (CA) of 97.6°, and a water solubility (WS) of 18.75%. The inclusion of plasticizers altered the crystalline structure of the CL matrix, smoothing and flattening the film surface while reducing hydrogen-bonding interactions. These structural changes led to a more uniform distribution of amorphous chain segments and a decrease in glass transition temperatures. Among the tested plasticizers, GLY exhibited the highest compatibility with CL, resulting in the smoothest surface morphology and delivering the most effective plasticizing effect. The CL-GLY film showed a dramatic improvement in flexibility, with an elongation at break that was 5.2 times higher than that of the unplasticized film (increasing from 5.39% to 33.14%), indicating significant enhancement in extensibility. Overall, these findings highlight the potential of CL-GLY films as sustainable and effective materials for food packaging applications.

## 1. Introduction

Most packaging materials are petroleum-based and non-degradable, contributing to “white pollution”. To mitigate packaging waste, there is a push to explore bio-based, renewable, and biodegradable materials for packaging. Edible materials like carbohydrates and proteins, which are renewable, biodegradable, and safe for food contact, represent a promising category of food packaging. Carbohydrate-based polymers, in comparison to protein-based polymers, contain simpler chemical structures without amino or carboxyl groups, giving them superior chemical stability and making them ideal for packaging [1]. However, these materials often exhibit high water solubility and poor water resistance, limiting their applications.

Curdlan (CL) is a glucose-based extracellular polysaccharide connected by β-1,3 glycosidic bonds. Produced through glucose fermentation by Alcaligenes faecalis var. myxogenes 10C3, CL is tasteless, colorless, odorless, insoluble in water and alcohol, but soluble in alkali. In water dispersions, CL can form thermoreversible gels (low-set) at high temperatures and thermoirreversible gels (high-set). Due to its unique physicochemical properties and rheological behavior, CL has become increasingly important in both food and non-food industries. However, the limited mechanical properties and water resistance of CL films restrict broader applications [2]. Non-volatile plasticizers such as polyols, including glycerol (GLY), sorbitol (SOR), polyethylene glycol (PEG), xylitol (XY), propylene glycol (PRO), and ethylene glycol (EG), possess good water solubility and compatibility with bio-based materials, making them effective plasticizers that enhance film extensibility [3]. Plasticizers increase the intermolecular distance and free volume, reducing intermolecular forces, and enhancing chain mobility, thus improving flexibility.

Plasticizer effectiveness depends on factors like molecular weight, polarity, and compatibility with the matrix. Studies have shown that glycerol’s lower molar mass made it more effective than sorbitol in plasticizing films (Rodríguez et al.) [4], while Tong et al. reported varied effects of glycerol, sorbitol, xylitol, and fructose on mechanical properties, barrier performance, and moisture resistance of amylopectin-alginate-carboxymethyl cellulose films [5]. Studies also observed that sorbitol-plasticized starch/PVOH/chitosan films exhibited the highest elongation at break, the lowest WVP, and improved heat resistance over films plasticized with GLY and PEG. Further, it was found that sorbitol increased elongation at break more effectively than glycerol, fructose, and urea in wheat starch films [6].

Despite various findings, no systematic research has examined the plasticization of curdlan films in detail. This study aims to systematically analyze the plasticizing effects of low-molecular-weight polyols (GLY, EG, PRO, XY, SOR, PEG) with different structural characteristics on CL-based films, focusing on molecular weight, structure, and compatibility. SEM, XRD, and FTIR techniques will assess morphology and microstructural changes in the films. Tensile testing will evaluate mechanical properties, while WVP, water solubility (WS), swelling ratio, and water contact angle (CA) tests will measure water sensitivity in the CL-based films.

## 2. Materials and Methods

### 2.1. Materials

Curdlan (CL, food grade) was purchased from Jiangsu Yiming Biological Tech-nology Co., Ltd. (Taizhou, China). Glycerol (GLY, analytical grade), ethylene glycol (EG, analytical grade), propylene glycol (PRO, analytical grade), xylitol (XY, analytical grade), sorbitol (SOR, analytical grade), and polyethylene glycol 400 (PEG 400, analytical grade) were obtained from China National Pharmaceutical Group Corporation (Sinopharm Chemical Reagent Co., Ltd., Shanghai, China). The physical properties of varying plasticizers are exhibited in Table 1.

### 2.2. Preparation of CL Films

**Scheme 1:**
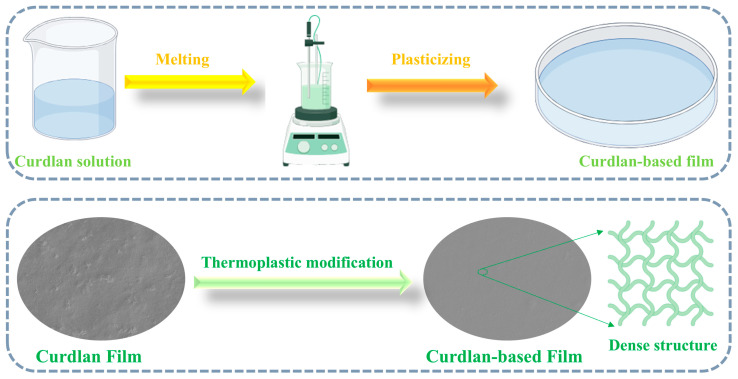
Diagram of the fabrication of CL films.

To prepare the curdlan (CL) films, CL was initially dispersed in distilled water and stirred for 30 min. Subsequently, 3 M of NaOH was added, and the mixture was stirred for an additional hour. The pH of the solution was then neutralized using 1 M of HCl, yielding a gel-like substance. This gel was centrifuged at 8000× *g* for 5 min to separate the supernatant, which was discarded. The remaining gel was washed with distilled water, repeating the centrifugation step four times to ensure purity. Following centrifugation, the solid content of the CL gel was adjusted to 1% (*w*/*w*) with distilled water. The gel was homogenized at 10,000 rpm for 5 min to obtain a film-forming solution. A plasticizer (10% of dry matter) was then added, and the mixture was stirred using a magnetic stirrer for 30 min, followed by vacuum degassing for 1 h to remove any trapped air. Approximately 70 g of the prepared solution was poured into a 15 cm diameter polystyrene Petri dish and dried in an oven at 37 °C for 18 h (as shown in Scheme 1). After drying, the film was peeled off and stored in a desiccator containing saturated NaBr solution for 48 h to equilibrate moisture content at 57% RH. The film prepared without a plasticizer was designated as the CL film, while films with glycerol (GLY), ethylene glycol (EG), propylene glycol (PRO), xylitol (XY), sorbitol (SOR), and polyethylene glycol (PEG) as plasticizers were designated as CL-GLY, CL-EG, CL-PRO, CL-XY, CL-SOR, and CL-PEG films, respectively.

### 2.3. Characterization of Film Properties

#### 2.3.1. SEM Morphology of the CL Films

The surface and cross-sectional morphology of the films were observed using a Zeiss field emission scanning electron microscope (Gemini SEM 300, Jena, Germany). The surface images were obtained at a voltage of 5 Kv, while the cross-sectional images were obtained at a voltage of 2 Kv. For cross-sectional samples, film specimens measuring 7 cm × 5 mm were fractured in liquid nitrogen to obtain cross-sections. These sections were then mounted on a sample holder, sputter coated with gold, and observed at 2000× magnification [7]. For surface samples, the films were cut into 1 cm × 1 cm pieces, directly mounted on the sample holder, sputter coated with gold, and observed at a magnification of 500×.

#### 2.3.2. Thickness and Appearance

The appearance of the films was captured using a digital camera, and their thickness was measured at seven different points on the film surface with a high-precision digital micrometer. The average thickness was then calculated, with each sample tested five times.

#### 2.3.3. Fourier Transform Infrared Spectroscopy (FTIR)

To investigate potential interactions between starch and plasticizers, a Cary 610 FTIR spectrometer (Santa Clara, CA, USA) was used. The testing conditions included a scanning range from 4000 cm^−1^ to 400 cm^−1^, a resolution of 4 cm^−1^, and 32 scans [8].

#### 2.3.4. X-Ray Diffraction (XRD)

Wide-angle XRD testing was conducted using a D8 Advance X-ray diffractometer. The testing conditions included monochromatic Cu-Kα radiation with a wavelength of 0.154 nm, an operating voltage of 40 kV, a current of 40 mA, and a scanning angle range from 3° to 40°, with a continuous scan rate of 3°/min [9].

#### 2.3.5. Dynamic Thermomechanical Analysis (DMA)

The dynamic thermomechanical properties of the films were analyzed using a DMA Q800 dynamic mechanical analyzer (TA Instruments, New Castle, DE, USA). Samples were pre-cut into strips measuring 3 cm × 2 mm [10]. The testing conditions included a temperature range of 25−300 °C, a frequency of 1 Hz, an amplitude of 5 μm, a heating rate of 2 °C/min, and a strain of 0.07%.

#### 2.3.6. Light Transmittance

A 759S UV-Vis spectrophotometer (Inesa, Shanghai, China) was used to analyze the film samples. Rectangular strips measuring 10 mm × 40 mm were placed in quartz cuvettes and scanned across a wavelength range of 200–800 nm [11]. Each sample was tested three times for accuracy.

#### 2.3.7. Contact Angle (CA)

Film samples were cut into 7 cm × 2 cm strips and mounted on glass slides, which were placed on the moving platform of a ZJ-7000 contact angle meter (Zhijia Instrument Equipment Co., Ltd., Shenzhen, China). A 5 μL drop of ultrapure water was applied to the film surface using a micro-syringe, and a photograph was taken upon contact. The contact angle was calculated using software fitting, and each sample was tested three times for accuracy [12].

#### 2.3.8. Swelling Ratio

The film was cut into 5 cm squares, and its initial weight was recorded. It was then continuously stirred in 50 mL of distilled water for 1 h, removed promptly, and blotted with filter paper to remove surface water [13]. The weight after removing the surface water was recorded, and the swelling ratio was calculated using the corresponding formula:

(1) $$\mathrm{Swelling}\;\mathrm{ratio}\;\left(\%\right)=\frac{\left(M_{i}-M_{f}\right)}{M_{i}}\times 100$$

Mi, the initial dry weight of the film; M_f_, the dry weight of the film after swelling and removing surface moisture. Each sample was tested 3 times.

#### 2.3.9. Water Solubility (WS)

For the water solubility test, the film was cut into 5 cm squares, dried to a constant weight at 105 °C, and the initial weight was recorded as M_i_. The film was stirred continuously in 50 mL of distilled water for 1 h, then the undissolved portion was removed, dried to a constant weight at 105 °C, and recorded as M_f_. The WS was then calculated using the appropriate formula:

(2) $$\mathrm{WS}\left(\%\right)=\frac{\left(M_{i}-M_{f}\right)}{M_{i}}\times 100$$

M_i_, the initial dry weight of the film; M_f_ the final dry weight of the undissolved film. Each sample was repeated 3 times, and the average was calculated.

#### 2.3.10. Water Vapor Permeability (WVP)

For water vapor permeability testing, film samples were cut into 7 cm squares and sealed onto centrifuge tubes containing 40× *g* of anhydrous silica gel. These tubes were placed in a desiccator containing distilled water at 20 °C [14]. The weight of the centrifuge tubes was recorded every 24 h for 7 consecutive days, and the water vapor permeability was calculated using the specified formula:

(3) $$\mathrm{WVP}=\frac{W\times x}{t\times A\times ∆P}$$

W, the change in the mass of the centrifuge tube (g), x, the thickness of the film (mm), t the time taken for the mass change (s), A the permeation area (m^2^), and ΔP the water vapor pressure difference (3186 Pa at 20 °C) across the two sides of the sample. Each sample was tested 3 times.

#### 2.3.11. Tensile Properties

Film samples, prepared as 60 mm × 6 mm rectangular strips, were subjected to tensile testing using a universal testing machine to determine tensile strength (TS) and elongation at break (EAB). For each test, an initial gauge length of 40 mm and a crosshead speed of 5 mm/min were used [15]. To ensure data reliability, each sample was tested seven times, with the results providing insight into the mechanical properties of the CL films, particularly in response to different plasticizers.

### 2.4. Statistical Analysis

Data analysis and figure generation were performed using SPSS Statistics 25 (IBM Corp.). The results were expressed as mean ± standard deviation (SD), and one-way analysis of variance (ANOVA) was applied, followed by Duncan’s multiple range test for mean comparison. Values with different letters represent significant differences (*p* < 0.05), while values with the same letter indicate no significant differences (*p* > 0.05).

## 3. Results and Discussion

### 3.1. Appearance and Thickness of CL Edible Films

The appearance and the thickness of the CL edible films with various plasticizers are shown in Table 2. The CL film appears rough with low transparency, but after adding plasticizers, molecular chain entanglement is reduced, resulting in a more uniform film structure. The thickness of the CL edible films with plasticizers was increased after the introduction of plasticizers. The CL film had the smallest thickness of 33.46 μm, while the CL-GLY film had the largest (42.93 μm). Additionally, the CL-XY film, CL-SOR film, and CL-PEG film exhibited thickness values of 36.39 μm, 41.04 μm, and 37.78 μm, respectively. Different plasticizers affected the film thickness differently, with PEG 400 having the least effect and GLY the greatest. This is likely because the small molecular weight and strong hydrophilicity of glycerol allowed it to easily insert into the polymer network, forming a more robust and thicker film.

### 3.2. Crystalline Properties of the CL Edible Films

**Figure 1:**
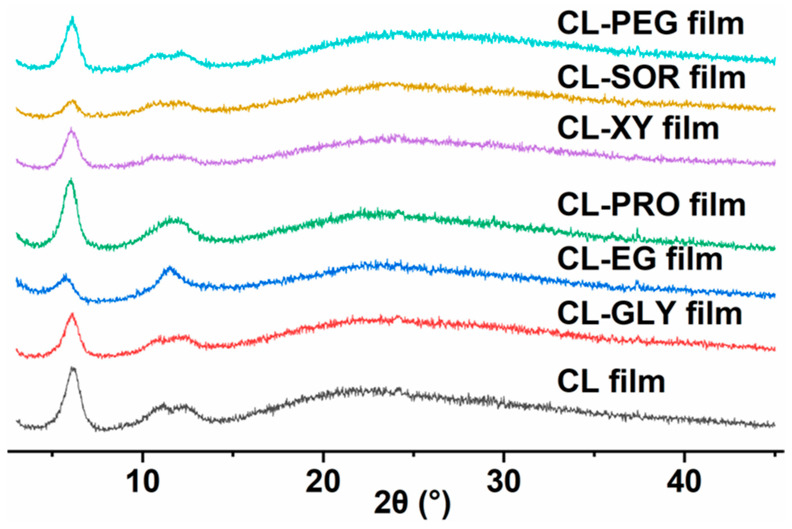
Wide-angle XRD pattern of CL edible film with different plasticizers.

The XRD patterns of CL edible films with different plasticizers are shown in Figure 1. The unplasticized CL film exhibited four peaks at approximately 6.2°, 11.1°, 12.2°, and 22.1°, with three corresponding to crystalline regions. The slight discrepancy in these results may be attributed to the higher water content in the CL films used in this study, as water can influence polymer crystallinity. The peak at 22.1° corresponds to the amorphous structure of the film. After the addition of GLY, EG, PRO, and SOR, the peak positions shifted slightly toward lower angles, and their intensities decreased. This suggests that the plasticizers disrupted the original arrangement of CL molecular chains, reducing intermolecular forces, increasing free volume between the chains, and thereby lowering crystallinity while enhancing chain mobility, which increases plasticity [16]. Upon incorporating PEG and XY, the crystalline peak at 6.2° shifted to a smaller angle, the peak at 12.2° disappeared, and a new peak appeared at 11.5°. These changes indicate the formation of new hydrogen bonds among PEG, XY, and CL, demonstrating enhanced compatibility and superior film-forming performance within the composite system. Overall, the crystallinity of the films decreased following the addition of plasticizers, with the most significant reduction observed in the PRO and SOR films. The interaction between the plasticizer molecules and CL weakens intramolecular forces, leading to reduced crystallinity.

### 3.3. SEM Morphologies of the CL Edible Film

**Figure 2:**
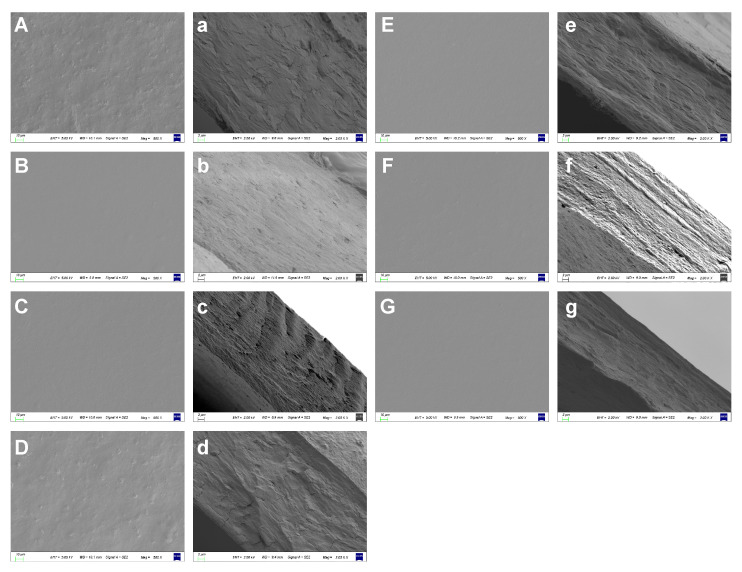
Surface and cross-sectional of CL edible film with different plasticizers. CL (**A**,**a**); CL-GLY (**B**,**b**); CL-EG (**C**,**c**); CL-PRO (**D**,**d**); CL-XY (**E**,**e**); CL-SOR (**F**,**f**); CL-PEG (**G**,**g**).

The SEM images of CL edible films with different plasticizers are presented in Figure 2. The surface of the unplasticized CL film appears rough and loosely structured. After the addition of plasticizers, all film surfaces became smoother, with the CL-GLY film exhibiting the smoothest surface. This suggests the best compatibility between GLY and CL, resulting in the most significant improvement in surface roughness [17]. The cross-section of the unplasticized CL film exhibited roughness and small cracks, consistent with the findings reported by the previous literature. However, following the incorporation of plasticizers, the cracks in the cross-sections disappeared, and the surface roughness was significantly reduced. Among the modified films, those containing GLY and PEG displayed the smoothest cross-sections, indicating excellent compatibility between these plasticizers and the CL matrix. In contrast, the cross-sections of CL films with EG, PRO, SOR, and XY appeared dense and uneven, characterized by prominent ridges and patterns likely resulting from strong internal bonding forces [18]. These characteristics are attributed to high internal cohesion during the slow drying process of the films.

### 3.4. FTIR of CL Edible Films

**Figure 3:**
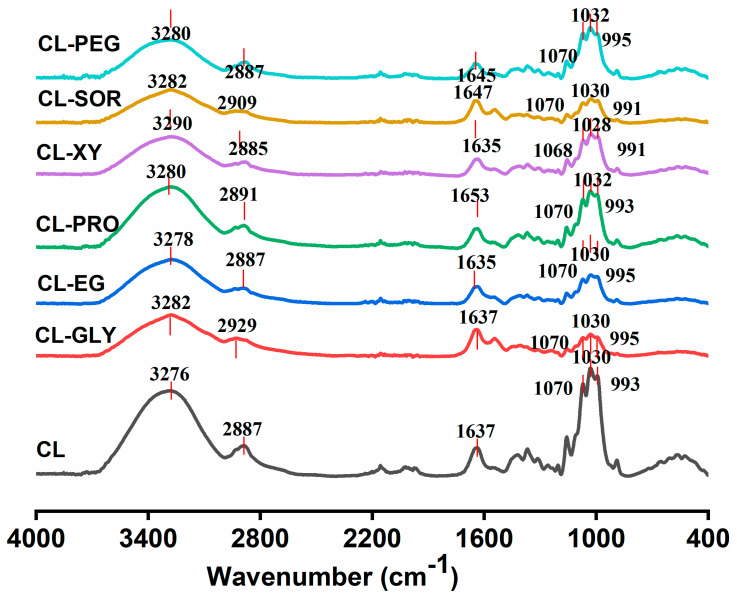
Fourier transform infrared spectrogram of CL edible film with different plasticizers.

The infrared spectra of CL edible films with various plasticizers are represented in Figure 3. The unplasticized CL film shows stretching vibration peaks at 991 cm^−1^, 1027 cm^−1^, and 1070 cm^−1^, corresponding to the C-O-C and C-O groups. A stretching vibration peak for free water (H-O-H) is observed at 1637 cm^−1^, and the stretching vibration peak of the -CH_3_ group is present at 2887 cm^−1^. Compared to the CL film, the CL-GLY film exhibits a shift in the -OH stretching vibration peak from 3282 cm^−1^ to 3276 cm^−1^, while the -CH_3_ stretching peak shifts from 2887 cm^−1^ to 2927 cm^−1^, and the C-O-C and C-O stretching peaks shift from 993 cm^−1^ to 995 cm^−1^. In the CL-EG film, the -OH stretching peak shifts from 3278 cm^−1^ to 3276 cm^−1^, the H-O-H stretching peak moves from 1637 cm^−1^ to 1635 cm^−1^, and the C-O stretching peaks shift from 993 cm^−1^ to 995 cm^−1^. For the CL-PRO film, the-OH stretching peak moves from 3280 cm^−1^ to 3276 cm^−1^, the H-O-H stretching peak shifts from 1653 cm^−1^ to 1637 cm^−1^, and the C-O stretching peaks move from 1030 cm^−1^ to 1032 cm^−1^. In the CL-XY film, the -OH stretching peak shifts from 3276 cm^−1^ to 3290 cm^−1^, the -CH_3_ stretching peak moves from 2887 cm^−1^ to 2885 cm^−1^, and the H-O-H stretching peak shifts from 1637 cm^−1^ to 1635 cm^−1^. In the CL-SOR film, the -OH stretching peak moves from 3276 cm^−1^ to 3282 cm^−1^, the -CH_3_ stretching peak shifts from 2887 cm^−1^ to 2909 cm^−1^, the H-O-H stretching peak moves from 1637 cm^−1^ to 1647 cm^−1^, and the C-O-C and C-O stretching peaks shift from 993 cm^−1^ to 991 cm^−1^. Lastly, in the CL-PEG film, the -OH stretching peak shifts from 3276 cm^−1^ to 3280 cm^−1^, the H-O-H stretching peak moves from 1637 cm^−1^ to 1645 cm^−1^, and the C-O-C and C-O stretching peaks shift from 1030 cm^−1^ to 1032 cm^−1^. The incorporation of plasticizers resulted in a slight blue shift in the -OH stretching vibration peak, suggesting a redistribution of hydrogen bonds and a reduction in interactions between plasticizer molecules and the CL film, which aligns with the XRD findings [19]. Additionally, the peaks associated with C-O-C, C-O, and -CH_3_ groups also shifted, indicating an impact on hydrophobic interactions within the system.

### 3.5. Thermal Properties

**Figure 4:**
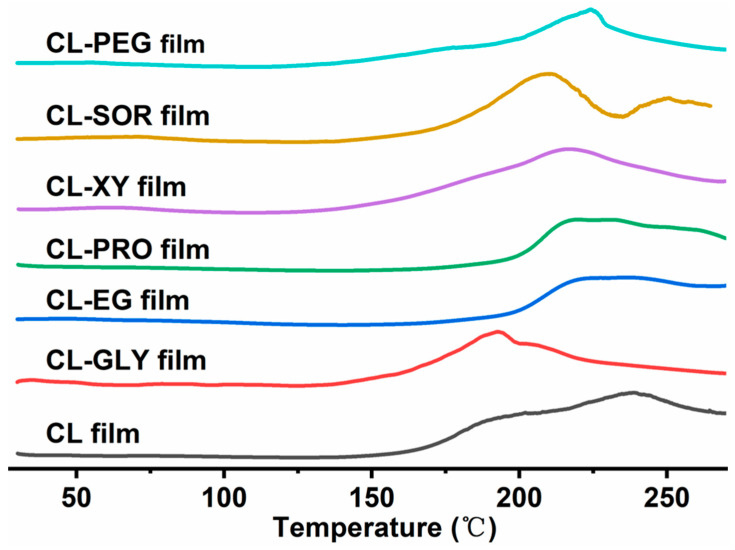
Dynamic thermomechanical properties of CL edible films with different plasticizers.

The dynamic mechanical properties of CL edible films with various plasticizers are presented in Figure 4. The temperature corresponding to the maximum value of the tan δ peak represents the glass transition temperature (Tg). After the incorporation of plasticizers, the Tg of the films decreases, as shown in Table 3. The pure CL film exhibits three distinct transition peaks, indicating significant variability in the binding degree of the molecular chains in the amorphous regions, suggesting a heterogeneous system. With the addition of plasticizers, the number of peaks decreases, implying that the plasticizers reduce the variability in the binding of the amorphous chains, leading to a more homogeneous system [20]. This observation aligns with the SEM results. Both the CL-GLY and CL-PEG films display one prominent transition peak and a smaller secondary peak, suggesting good compatibility between GLY, PEG, and CL. In contrast, the CL-XY and CL-SOR films show two pronounced transition peaks, while the transition peaks in the CL-PRO and CL-EG films broaden, appearing as a superposition of multiple peaks. This broadened glass transition region suggests heterogeneity, indicating that while XY, SOR, PRO, and EG demonstrate some compatibility with the CL system, their effect is limited. This finding is consistent with the XRD results. Tg is influenced by molecular structure, intermolecular interactions, and structural rigidity. The decrease in Tg is attributed to the plasticizers’ low molecular weight, which reduces the polymer’s glass transition temperature. The small size and low molecular weight of the plasticizers allow them to be distributed throughout the matrix, forming polar attractions with CL chain segments [21]. This interaction lowers the Tg and enhances the mobility of the chain segments, resulting in improved flexibility and processability of the matrix.

### 3.6. Light Transmittance of the Starch Films

**Figure 5:**
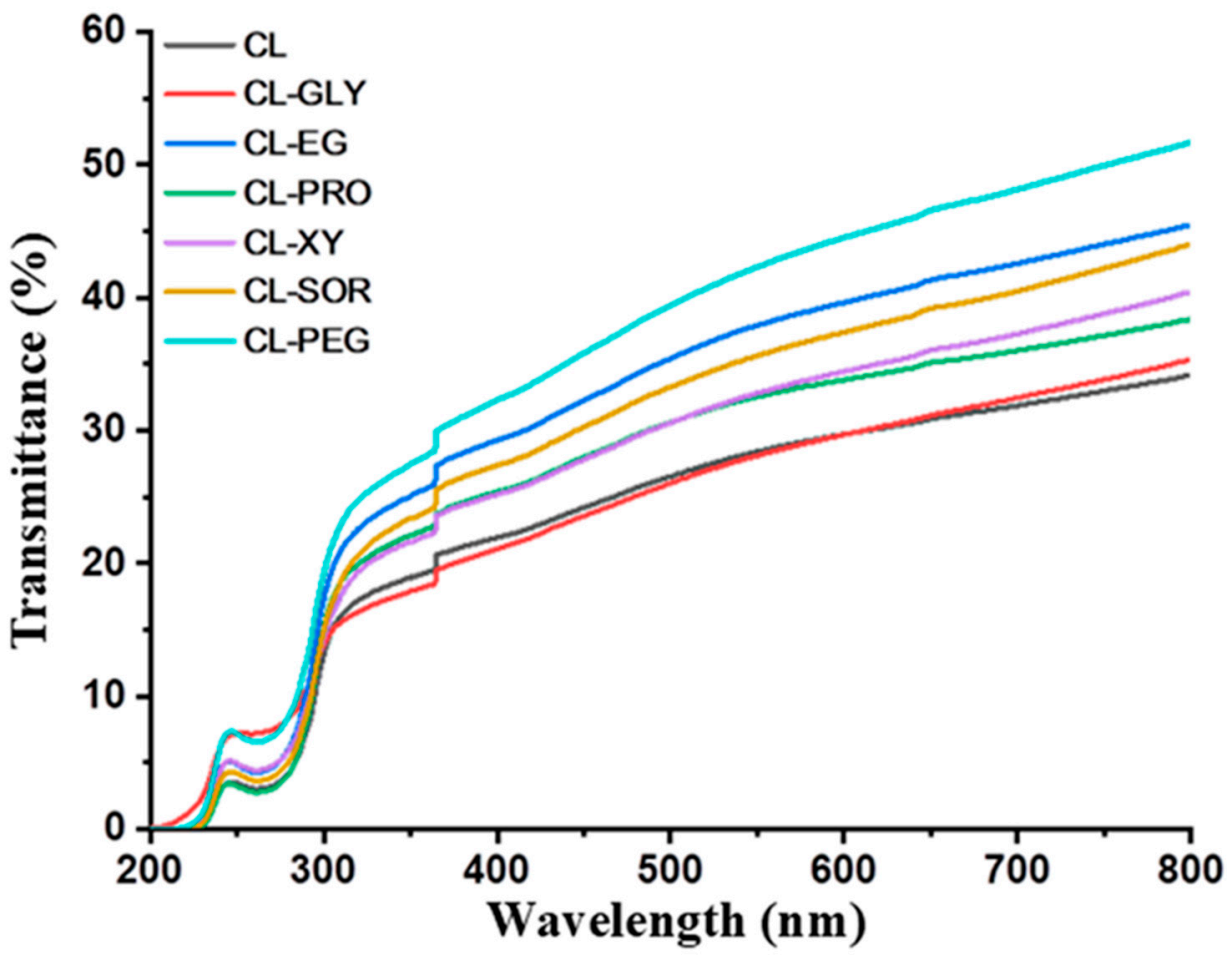
Light transmittance diagram of CL edible films with different plasticizers.

The light transmittance of CL edible films with various plasticizers is presented in Figure 5. The ranking of light transmittance from highest to lowest is as follows: CL-PEG > CL-EG > CL-SOR > CL-XY > CL-PRO > CL-GLY > CL film. The addition of plasticizers led to an increase in light transmittance to varying extents. Factors such as crystalline structure, surface morphology, and moisture content influence the films’ light transmittance [22]. Generally, a denser crystalline structure and higher crystallinity result in lower light transmittance. In contrast, a smoother and more uniform surface improves compatibility, leading to higher light transmittance. Additionally, films with higher moisture content exhibit better light transmittance. The high light transmittance of the PEG-containing film is primarily attributed to its low crystallinity and smooth surface [23]. Similarly, the EG-containing film shows elevated light transmittance; although it has higher crystallinity, its increased moisture content likely contributes to this improvement. The film with SOR also displays relatively high transmittance due to its low crystallinity. Although the XY-containing film has higher crystallinity than the other plasticized films, its smooth surface and high moisture content enhance its light transmittance [24]. The PRO-containing film has a rougher surface compared to other plasticized films, yet its low crystallinity and high moisture content still result in increased light transmittance.

### 3.7. Hydrophobicity of the Films

**Figure 6:**
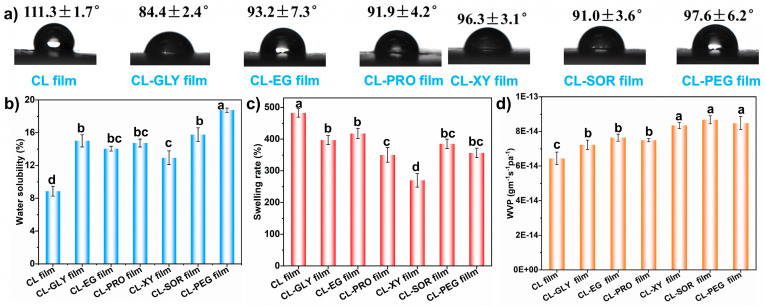
Hydrophobicity of the CL films. (**a**) CA; (**b**) WS; (**c**) swelling ratio; (**d**) WVP. The values were reported as Mean ± SD and statiscal significance is denoted by different letters (*p* < 0.05, Tukey’s test in one-way ANOVA).

The CA of CL edible films with various plasticizers is shown in Figure 6a. CA is a key parameter for assessing wettability and surface hydrophobicity, with a water CA of 90° typically serving as the threshold for defining hydrophilicity versus hydrophobicity. The addition of plasticizers reduces the surface hydrophobicity of CL films due to the hydrophilic nature of the plasticizers, which increases the film’s affinity for water. The distinct trend in the CA of PRO-containing films may result from their lower WVP compared to XY- and EG-containing films, leading to higher hydrophobicity. Reducing surface roughness also decreases the CA of the films. The lowest CA is observed in the CL-GLY film, with a value of 84.4°, likely due to its smoother surface [25]. Overall, the results indicate that the surface hydrophobicity of CL films decreases with the plasticizer addition, while CA values remain around 90°, thus preserving good water resistance.

The swelling ratio of CL edible films with various plasticizers is shown in Figure 6b. In order from highest to lowest, the swelling ratios are as follows: CL film > CL-EG film > CL-GLY film > CL-SOR film > CL-PEG film > CL-PRO film > CL-XY film. The addition of plasticizers significantly reduces the films’ swelling ratios, likely due to a decrease in -OH bonding and hydrogen bonding within the system after plasticization. The decrease in swelling ratio may be attributed to the increased crosslinking degree between CL molecules and plasticizers after plasticization, which enhances retraction forces and leads to a reduction in swelling. As a result, the interactions among CL molecules weaken, reducing the crosslinking degree within the network [26]. This, in turn, diminishes the network’s structural strength and elasticity, thereby lowering the swelling ratio.

The WS of CL edible films with different plasticizers is shown in Figure 6c. Solubility serves as an indicator of a film’s water resistance. All plasticizer-containing CL films demonstrate significantly higher solubility than the pure CL film, with the CL-PEG film showing the highest WS at 18.75%. Due to the hydrophilic nature of the plasticizers, their leaching into water raises the solubility of the plasticized films. As the concentration of plasticizers increases, film solubility also rises. Plasticizers reduce the intermolecular interactions among polymers, increasing the interaction sites between the film’s molecular chains and water molecules, which enhances water affinity and results in higher solubility [27].

The WVP of CL edible films with different plasticizers is shown in Figure 6d. Adding plasticizers to CL films raises the water vapor permeability (WVP), solubility, and moisture content while reducing the swelling ratio. The increase in WVP and moisture content after the plasticizer addition may be the result of the high -OH group content in the plasticizers, which enhances hydrogen-bonding interactions with water, leading to greater hygroscopicity and a higher effective diffusion coefficient for water vapor within the film [28]. Consequently, the WVP of the film rises. The increase in solubility occurs because plasticizers reduce intermolecular interactions among polymer chains and increase interaction sites between polymer chains and water molecules, thus elevating the polymer’s affinity for water and increasing solubility.

### 3.8. Tensile Properties of the CL Films

**Figure 7:**
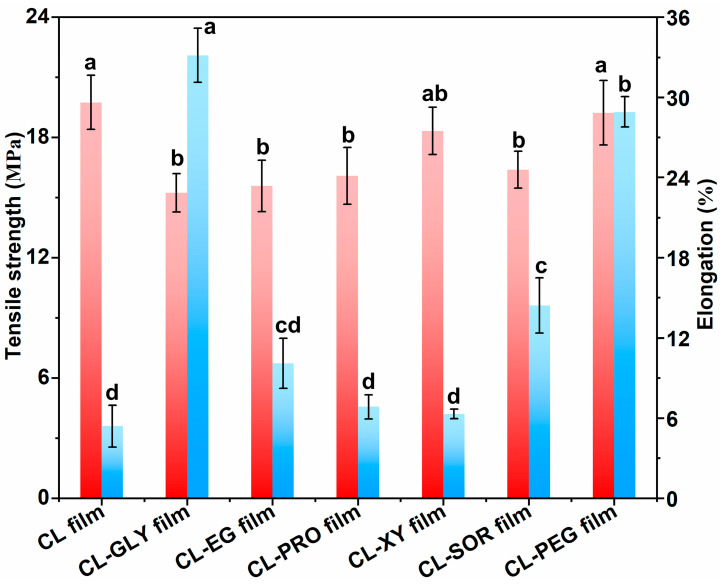
Mechanical properties of CL edible film with different plasticizers. The values were reported as Mean ± SD and statiscal significance is denoted by different letters (*p* < 0.05, Tukey’s test in one-way ANOVA).

Figure 7 shows the mechanical properties of CL edible films. Tensile strength (TS) represents the maximum tensile stress a film can withstand, while the elongation at break (EAB) refers to the maximum change in length before the sample breaks. The tensile strength from highest to lowest is as follows: CL-GLY film > CL-PRO film > CL-EG film > CL-XY film > CL-PEG film > CL film > CL-SOR film. The TS value of the unplasticized film is 19.74 MPa, and the EAB is 5.39%. The addition of plasticizers decreases the TS and increases the EAB of CL films. This is attributed to the plasticizers significantly reducing the intermolecular forces in CL, leading to an increase in chain mobility and free volume within the polymer matrix [29]. The intermolecular and intramolecular interactions in the polymer are weakened, and interactions between the plasticizer and polymer are formed, resulting in lower tensile strength and higher elongation at break. The CL-GLY film exhibited the best mechanical properties, with the highest EAB of 33.14% and a TS of 15.23 MPa. This may be due to the molecular size, configuration, and total number of functional hydroxyl groups of the plasticizer, as well as its compatibility with the polymer, which influence the interactions between the plasticizer and polymer. Compared to other polyols, the poorer plasticization effect of PRO might be due to the presence of hydrophobic-CH3 groups in its molecular structure, which reduce compatibility with the CL system [2,30]. In contrast, glycerol has a smaller molecular size and more -OH groups, which improve its compatibility with the CL system, enhancing its effectiveness as a plasticizer. Therefore, GLY shows better plasticization effects [31].

## 4. Conclusions

This study investigated the effects of various plasticizers—glycerol (GLY), ethylene glycol (EG), propylene glycol (PRO), xylitol (XY), sorbitol (SOR), and polyethylene glycol (PEG)—on the structural and physical properties of curdlan (CL) edible films. The results revealed that plasticizers modified the crystalline structure of the films, with PEG and XY inducing shifts in crystalline peaks and altering the arrangement of the crystalline matrix. These modifications enhanced surface smoothness and reduced both the number and temperature of glass transition peaks. Notably, CL-GLY films exhibited exceptionally smooth surfaces and cross sections, demonstrating strong compatibility between GLY and CL. The addition of plasticizers increased the transparency, water vapor permeability (WVP), and water solubility of the films, although a moderate rise in water sensitivity was observed. Despite this, the films retained satisfactory water resistance. Among the plasticizers, GLY significantly enhanced the mechanical properties, producing films with reduced tensile strength but the highest elongation at break, reflecting increased flexibility. Overall, GLY was identified as the most effective plasticizer, creating films with balanced mechanical performance and water resistance. These attributes highlight the potential of GLY-plasticized CL films as sustainable materials for food packaging applications.

## Figures and Tables

**Table 1:** Types and properties of plasticizers.

Plasticizers	M (g/mol)	Density (g/cm^3^)	X ^a^	Y ^b^	Melting Point (°C)
SOR	182	1.49	6	1.39 × 1021	88–102
XY	152	1.52	5	1.38 × 1021	94–97
GLY	92	1.30	3	1.37 × 1021	17.4
PRO	76	1.04	2	1.10 × 1021	−59
EG	62	1.11	2	1.36 × 1021	−12.9
PEG 400	380–430	1.27	1	9.80 × 10^19^–1.11 × 10^20^	64–66

X ^a^ shows the number of OH in a single molecule; Y ^b^ indicates the number of hydrogen nodes in molecules under unit mass.

**Table 2:** Thickness of CL edible film with different plasticizers.

Sample	CL	CL-GLY	CL-EG	CL-PRO	CL-XY	CL-SOR	CL-PEG
Appearance	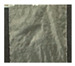	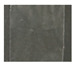	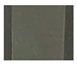	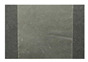	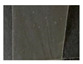	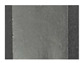	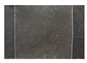
Thickness (μm)	33.46 ± 1.15 ^c^	42.93 ± 1.11 ^a^	41.47 ± 0.62 ^a^	40.38 ± 1.42 ^a^	36.39 ± 1.28 ^b^	41.04 ± 0.92 ^a^	37.78 ± 1.26 ^b^

The values were reported as Mean ± SD and statiscal significance is denoted by different letters (*p* < 0.05, Tukey’s test in one-way ANOVA).

**Table 3:** Glassy transition temperature of CL edible film with different plasticizers.

	CL	CL-GLY	CL-EG	CL-PRO	CL-XY	CL-SOR	CL-PEG
Peak	237.8	191.8	232.3	227.8	216.4	214.2	223.3
Tg (°C)	±3.4 ^a^	±1.4 ^d^	±2.1 ^a^	±2.1 ^b^	±0.7 ^c^	±1.7 ^c^	±1.3 ^b^

The values were reported as Mean ± SD and statiscal significance is denoted by different letters (*p* < 0.05, Tukey’s test in one-way ANOVA).

## Data Availability

The original contributions presented in this study are included in the article. Further inquiries can be directed to the corresponding authors.

## Abbreviations

CL, Curdlan; GLY, Glycerol; EG, Ethylene glycol; PRO, propylene glycol; XY, Xylitol; SOR, sorbitol; PEG, polyethylene glycol; FTIR, Fourier transform infrared spectroscopy; XRD, X-ray diffraction; SEM, scanning electron microscopy; DMA, dynamic mechanical analysis; WVP, water vapor permeability; CA, contact angle; WS, water solubility; UV-vis, ultraviolet-visible; TS, tensile strength; EAB, elongation at break; RH, relative humidity; SD, standard deviations; Spass, Statistical Program for Social Sciences.
